# Flock health survey of Irish Texel society breeders and larynx examination in Texel sheep

**DOI:** 10.1186/s13620-020-00170-2

**Published:** 2020-08-07

**Authors:** Aideen Kennedy, Séamus Fagan, Colm Brady, John Fagan, Eamon Wall, Seamus Hoey, Emma Tobin, Mícheál Casey, Maresa Sheehan

**Affiliations:** 1 Department of Agriculture, Kilkenny Regional Veterinary Laboratory, Food and the Marine, Kilkenny, Ireland; 2 Department of Agriculture, Athlone Regional Veterinary Laboratory, Food and the Marine, Athlone, Westmeath Ireland; 3 Sheep Ireland, Bandon, Co, Cork Ireland; 4 Diagnostic Imaging and Anaesthesia, School of Veterinary Medicine, University College Dublin, Dublin, Ireland; 5 Department Agriculture, Regional Veterinary Laboratories Division, Backweston Campus, Food and the Marine, Celbridge, Kildare Ireland

**Keywords:** Flock health, Laryngeal chondritis, Larynx, Ovine, Pathology, Texel

## Abstract

**Background:**

Laryngeal chondritis is a disease of undetermined aetiology, characterised by oedema, ulceration, abscessation and necrosis of the laryngeal mucosa and cartilage. The initial aim of the study was to document flock health issues identified by Irish pedigree Texel breeders using a questionnaire survey. Additionally, given the reports of breed predisposition for laryngeal chondritis in Texels, a further aim was to identify if laryngeal problems were perceived as an issue. Work was then conducted to identify if pre-clinical laryngeal mucosal pathology was identifiable in Texel sheep showing no overt clinical signs of respiratory disease and if associations existed between laryngeal measurements and laryngeal pathology.

Thirty one larynges were collected from a Texel flock that previously had laryngeal chondritis diagnosed in fallen stock. Gross visual inspection was performed to identify and grade (0–5) laryngeal pathology. A series of measurements were then performed on larynges that had been formalin fixed. Associations between independent variables (larynx measurements) and the dependent variable (laryngeal pathology score) were examined.

**Results:**

Respiratory disease was the most frequently identified health issue. Farmer-diagnosed ‘throat problems’ were reported by over 80% of respondents.

Laryngeal pathology was noted in Texels showing no overt clinical signs of respiratory disease. Associations between laryngeal measurements and laryngeal pathology were identified relating to the angle between the cranial point of the cricoid cartilage and the vocal process of the arytenoid cartilage.

**Conclusions:**

Mild laryngeal pathology was noted in animals with no overt clinical signs of respiratory disease. Future research should examine whether significant associations between laryngeal measurements and laryngeal pathology identified in the current study can be measured ante mortem, and whether such ante mortem measurements will allow early identification of sheep at risk of developing laryngeal chondritis.

## Background

**Fig. 1:**
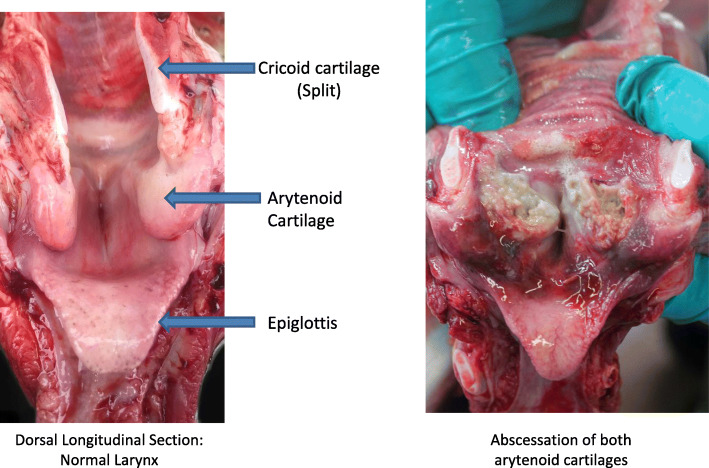
Dorsal view of longitudinal section of **a**) normal larynx and upper trachea **b**) abscessation of both arytenoid cartilages

The larynx, the connection between the pharynx and trachea, is a complex structure that facilitates respiration, prevents aspiration, and is the main organ of vocalization. The larynx is composed of the epiglottic, cricoid, thyroid, and paired arytenoid cartilages [1]. Across numerous species laryngeal pathology has been noted, including cattle, sheep, horses and dogs [2–4]. Laryngeal chondritis is a disease of the larynx of undetermined aetiology It is characterised by oedema, ulceration, abscessation and necrosis of the laryngeal mucosa and cartilage [5]. (Fig. 1). The condition is progressive [2] and typically fatal. Laryngeal chondritis has been reported in sheep [6–8], horses [2, 9] and cattle [3, 10]. In the case of sheep a predisposition of the Texel breed is suggested [7, 11, 12], although the condition is not exclusive to this breed [11, 13]. While the pathogenesis remains unclear, it is believed that damage to the laryngeal mucosa allows entry of pathogens, aiding the bacteria to gain access to deeper tissues, which leads to inflammation in the cartilage [8]. Reasons postulated as the cause of mucosal lesions include aspiration of grass awns, dosing gun injuries and repeated trauma of the larynx due to dyspnoea [6, 8, 14]. In a study of fallen stock, Waine et al., 2019, identified differences in laryngeal anatomy between Texel and Blue-faced Leicester breeds and suggests the anatomical differences could have a detrimental effect on the function of the larynx.

In a recent study of sentinel flocks in Ireland, Murray et al. [15] indicated the specific requirement for further research investigating laryngeal chondritis in Irish flocks.

Therefore the initial aim of this study was to document flock health issues identified by Irish pedigree Texel breeders and to identify if laryngeal problems were perceived as an issue by Irish Texel farmers. Based on the results of the initial survey, additional work was conducted with the aim of examining if laryngeal chondritis is identifiable in Texel sheep showing no clinical signs of respiratory disease. Furthermore, a series of measurements taken on all larynges aimed to identify if associations exist between such laryngeal measurements and laryngeal pathology.

## Methods

### Survey procedure

The Irish Texel Society is made up of over 370 pedigree Texel sheep farmers distributed across the 26 counties of the Republic of Ireland. The ‘Premier Sale’ is the main society sale each year, as such it was chosen as the location for survey distribution. Survey distribution was in hardcopy format during the sale. Participation was voluntary and non-incentivized. The survey could be completed anonymously, or if interested in partaking in future work, an option was available to include the flock-owner’s name and their Texel Society flock number.

### Survey questionnaire

Questions were compiled based on information gathered from peer-reviewed publications, and based on the experience of an expert group consisting of farmers, veterinarians, researchers and members of Sheep Ireland. Following piloting of the questionnaire by members of the expert group, a number of minor modifications improved the questionnaire prior to circulation. This included referring to laryngeal chondritis as ‘throat problems’ and using twin lamb disease as an example of metabolic disease. This was to facilitate understanding amongst survey participants. The final questionnaire consisted of 10 questions (Table 1) predominantly structured in closed format. Two questions had additional subsections if the “yes” option was selected. Seven was the maximum number of additional subsection questions (Question 8).

**Table 1:** Questionnaire

Question	Response Options	Response (%)
**Flock type**	Pedigree only	24.6
	Commercial and pedigree	75.4
**Pedigree flock size**	< 25 Breeding ewes	12.3
	25–50 Breeding ewes	24.6
	> 50 Breeding ewes	63.1
**Are pedigree lambs fed concentrates between 8 weeks and sale?**	Yes	93.8
	No	6.2
**If yes are they fed ad lib?**	Yes	21.3
	No	78.7
**What is the max daily feeding rate kg per day**		Range 0.5 to 3–4 kg
**Percentage of pedigree lambs sold as breeding stock annually?**	≥50% lambs	60%
	< 50% lambs	40%
**Do you have an unusually high proportion of unexplained sudden deaths?**	Yes	4.6
	No	95.4
**Have you had sheep with throat problems e.g. loud harsh breathing?**	Yes	81.5
	No	18.5
Average how many per year (% of flock)	Range from 1 to 10%. Some didn’t give % and ranged from rare to 5 sheep	
What age most commonly affected	< 6 months	3.8
	6–12 months	13.2
	> 1 year	81.1
Does it occur more in males than females	Yes	17.0
	No	77.4
Does it occur at certain times of year	Housing	3.8
	Breeding	5.7
	Near finishing	17.0
	No obvious pattern	58.5
	Lambing	1.9
Do you attempt treatment?	Yes	73.6
	No	26.4
Is treatment successful?	Yes	23.1
	No	17.95
	Sometimes	58.97
Have you seen this problem more frequently when you have used a particular sire?	Yes	37.7
	No	47.2
	Unsure	13.2
Have you had poor thrive in purebred texels you can’t explain?	Yes	18.5
	No	81.5
Do you submit unexplained deaths to Regional Vet Lab for post mortem?	Yes	32.8
	No	65.6
Interested in partaking in further work to improve Texel sheep health?	Yes	73.8
	No	26.2

### Descriptive analysis: survey

Hardcopy survey responses were entered into Microsoft Excel (MS Office, Version 2010). Responses were coded to organize the data and complete descriptive analysis.

Questionnaires were deemed suitable for analysis if greater than two thirds of the survey questions were answered.

### Larynx selection

Thirty one larynges were collected from a Texel flock that previously had laryngeal chondritis diagnosed in fallen stock referred by a veterinary practitioner for post mortem at Kilkenny Regional Veterinary laboratory (RVL). All animals were clinically well, showed no overt signs of respiratory disease prior to being selected for slaughter and all passed ante mortem factory inspection. All were less than 1 year of age. All had their carcass weight recorded at the factory.

### Post mortem examination

Larynges were harvested on the factory line. Following factory collection, all larynges were returned to Kilkenny RVL for post mortem examination (PME). Two Texel larynges were damaged at collection and were excluded from further analysis.

**Fig. 2:**
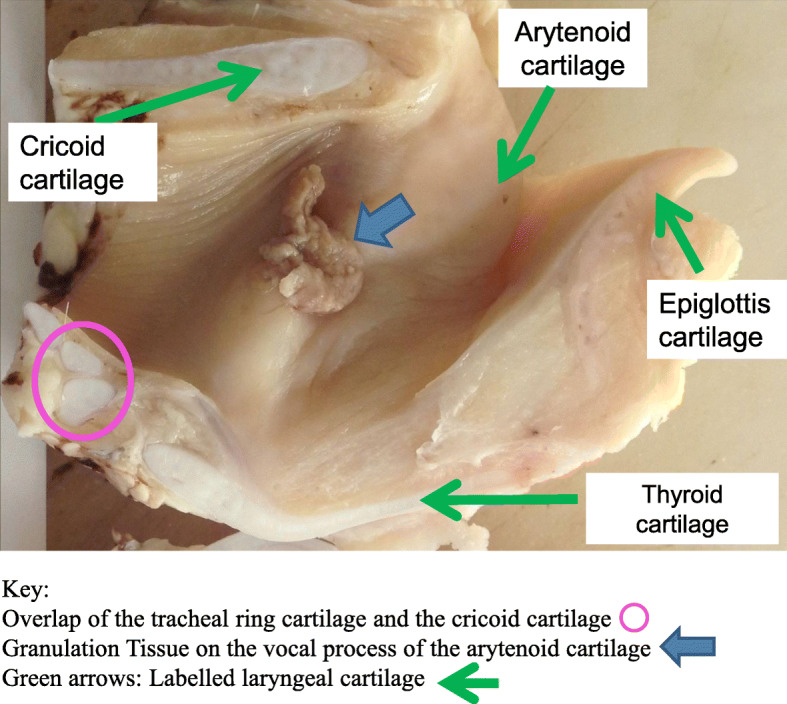
Sagittal section of ovine larynx (formalin fixed). Example of granulation tissue at the vocal process of the arytenoid cartilage. Overlap of the tracheal ring cartilage and cricoid cartilage shown in the pink circle

**Fig. 3:**
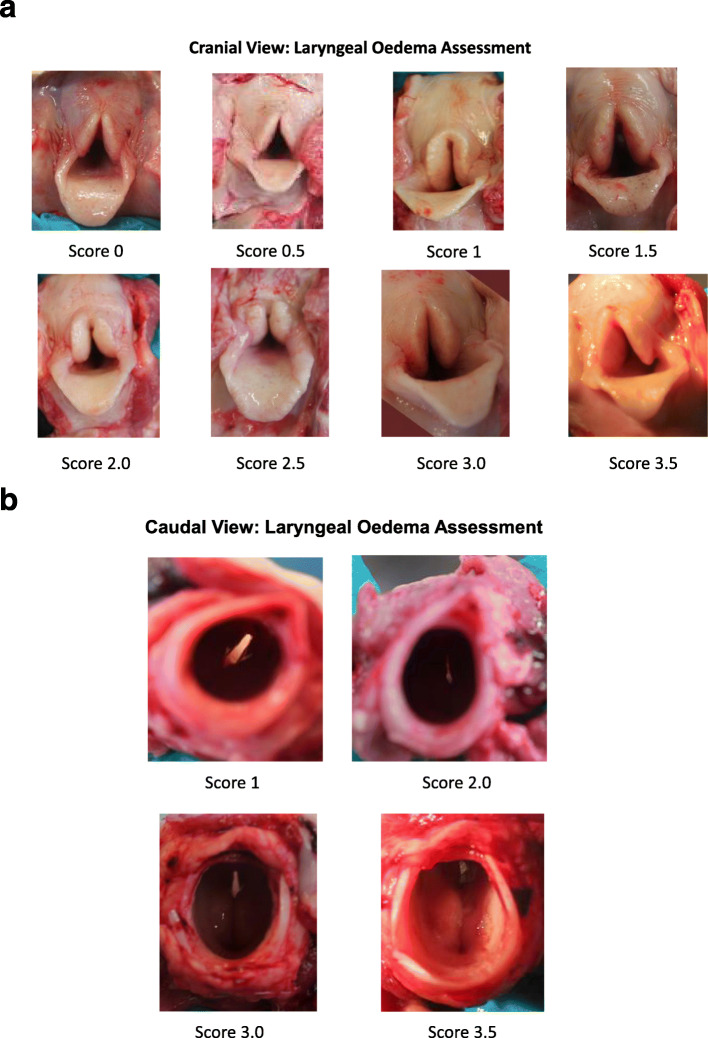
**a:** Scale of laryngeal oedema cranial view. It should be noted the assessment and subsequent score of each larynx was based on combining both cranial and caudal view. **b:** Scale of laryngeal oedema caudal view. On sectioning Larynx 3.5 granulation tissue was recorded in this larynx

Gross visual inspection allowed identification of laryngeal pathology. Assessment of the arytenoid mucosa included noting of oedema or inflammation. Oedema in the adjacent laryngeal soft tissue was included in the overall larynx assessment. Additionally if granulation tissue (Fig. 2) was identified, this was also recorded. Larynges were categorised as having mucosal pathology or not. Lesions were subsequently graded based on severity (0–5). Examination was conducted assessing both the cranial and caudal aspect of each larynx. Scoring was based on the overall impression of oedema and inflammation as assessed by three experienced pathologists. Mild oedema scored 0.5, with increasing score in increments of 0.5 for increasing level of oedema. Severe oedema or granulation tissue scored 3.5 (Fig. 3). Lesions consistent with deep ulceration, abscessation and necrosis of underlying laryngeal cartilage scored 4, 4.5 and 5 respectively. Fig. 3 b give an illustrated guide to scoring assessment.

### Fixed laryngeal examination

**Fig. 4:**
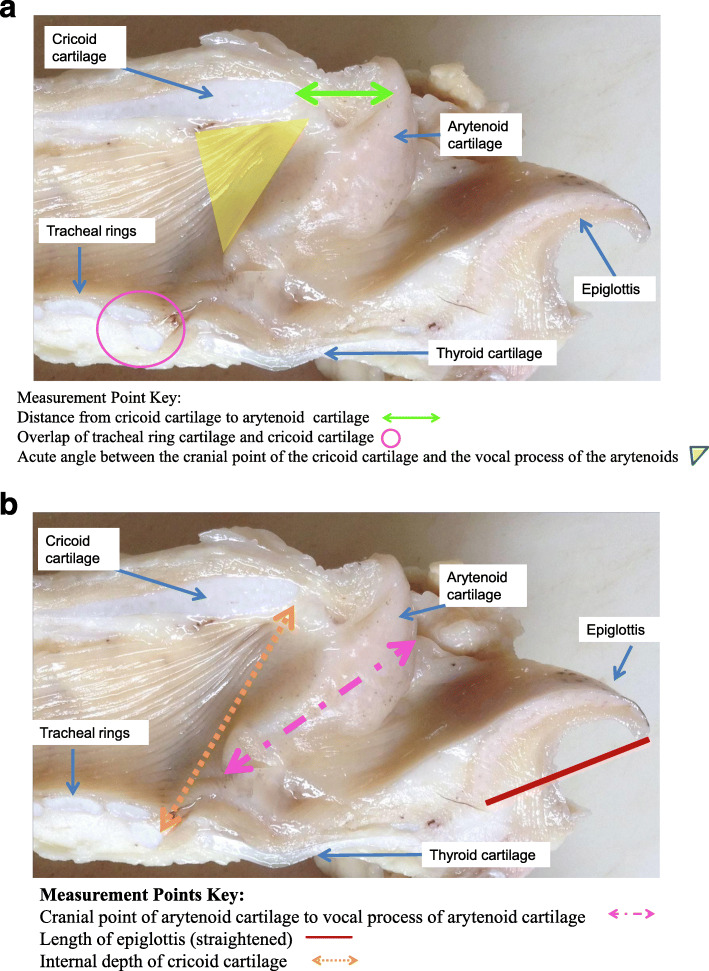
**a:** Sagittal section of ovine larynx (formalin fixed). Measurement points labelled include, distance from cricoid cartilage to the arytenoid cartilage, overlap of the tracheal cartilage and cricoid cartilage and acute angle between the cranial point of the cricoid cartilage and the vocal process of the arytenoid cartilage. **b**: Sagittal section of ovine larynx (formalin fixed). Measurement points labelled include the cranial point of the arytenoid cartilage to the vocal process of the arytenoid cartilage, the length of the epiglottis and the internal depth of cricoid cartilage

Following gross post mortem examination and grading of pathology, Texel larynges were placed in individual labelled containers containing 10% formalin and allowed to fix prior to further examination. Post-fixing a series of measurements was performed (Fixed Laryngeal Examination (FLE). On removal of each larynx from formalin, the left side was marked using a staple. Each larynx was then split centrally along the epiglottis. A series of six measurements was performed on both the left and right side of each split larynx- see table (Fig. 4 b). Tracheal overlap (Fig. 2) was categorised based on recording no overlap, the first tracheal ring covering less than half of the cricoid cartilage, the tracheal ring covering half of the cricoid cartilage, greater than 50% of the cricoid overlapped and complete overlap of the cricoid by the tracheal ring. The first tracheal ring was inadvertently removed by factory trimming on five of the larynges. Measurement of tracheal overlap was not possible on these five larynges; all other measurements however were conducted on the five larynges.

### Statistical analysis

Descriptive analysis and dataset construction were completed in Microsoft Excel (MS Office 2010). Multivariable linear regression was completed using Stata (version 12). Carcass weight was forced into all regression models as a fixed variable. The model examined associations between independent variables (larynx measurements) and the dependent variable- laryngeal pathology score (0–5). A manual backwards elimination with a forward step was performed for each model. Interactions between variables were also examined. Variables recording a significance level of *P* < 0.05 were retained in the model and are reported. As all samples were collected post mortem, the DAFM ethics committee deemed licensing unnecessary.

## Results

### Questionnaire

A total of 65 farmers returned questionnaires suitable for analysis, with a total of forty-one fully complete surveys. Three of the survey returns had two questions left unanswered, and the remainder had just one question unanswered. The majority of respondents had both pedigree and commercial flocks, with over 60% of respondents having more than 50 pedigree ewes in their flock (Table 1).

Respiratory disease was the most frequently identified flock health issue. A number of respondents identified multiple health issues on their farm. Twenty respondents reported two health issues, two respondents identified three flock health issues on their farms, with four flock health issues identified by a further two respondents (Tables 2 and 3). When the “other” option was selected, responses included lameness (*n* = 2), orf (*n* = 1), dystocia (*n* = 1) and one respondent reported no flock health issues.

**Table 2:** The most frequently identified health issues identified by survey participants

Respiratory disease	44.6%
Metabolic disease e.g. Twin lamb disease	43.1%
Mastitis	27.7%
Abortion	12.3%
Parasites	10.8%
Other	7.8%
Lamb diarrhoea	1.5%

When other was selected as option the ailments listed included: lameness =2, orf = 1, none = 1, dystocia = 1

**Table 3:** Flock health issues identified when more than one issue was selected

Number of health problems	Two health problems *n* = 20 flocks	Three health problems *n* = 2 flocks	Four health problems *n* = 2 flocks
	Respiratory and metabolic = 8	Abortion & parasites & metabolic = 1	Respiratory & abortion & parasites & metabolic =1
	Respiratory and mastitis = 4		
	Metabolic and mastitis = 2	Respiratory & abortion & mastitis = 1	Respiratory & abortion & parasites & mastitis =1
	Abortion and mastitis =1		
	Abortion and metabolic =1		
	Parasitic and metabolic =1		
	Parasitic and mastitis = 1		
	Respiratory and lame = 1		
	Lamb diarrhoea and metabolic = 1		

Over 80% of respondents reported ‘throat problems’, with a maximum with-in flock incidence estimate of 10% (range 1–10%). Some respondents (*n* = 10) didn’t provide a percentage of the flock affected and instead gave the number of sheep affected, with answers ranging from one/ two sheep to five sheep. One responded that it was a “rare” problem in their flock. ‘Throat problems’ were most commonly noted in animals aged over 1 year. Seventeen percent reported ‘throat problems’ to occur more frequently in males, and the majority of those surveyed noted no obvious pattern in disease occurrence. Although not listed as response options, seven respondents noted more ‘throat problems’ arising in summer, and one suggested weather.

Submission of carcasses to Department of Agriculture Food and the Marine (DAFM) Regional Veterinary Laboratories for post mortem examination was reported by 32.8%. A willingness to partake in future work, to aid improvement of Texel flock health was expressed by almost 75% of those surveyed.

### Post mortem findings

Average and median measurements recorded are reported in Table 4. Shape and size variations of the cricoid and first tracheal cartilage were observed within the breed. The first tracheal ring covered more than half of the cricoid in 12 Texels. The average internal depth of the cricoid was 36.3 mm. The average length of the Texel epiglottis was 2.7 cm.

**Table 4:** Texel laryngeal measurements

	Texel ***n*** = 29 Average	Texel ***n*** = 29 Median	
Length of epiglottis	2.7 cm	2.7	cm
Distance from cricoid to arytenoid- left	7.8	8.0	mm
Distance from cricoid to arytenoid- right	7.8	8.0	mm
Acute angle between the cranial point of the cricoid and the vocal process of the arytenoid right	56.5	55	°
Acute angle between the cranial point of the cricoid and the vocal process of the arytenoid left	58.1	60	°
Cranial point of arytenoid to vocal process of arytenoid left	28.1	28	mm
Cranial point of arytenoid to vocal process of arytenoid right	28	27	mm
Internal depth of cricoid	36.3	35	mm

#### Pathological lesions

All lesions were mucosal and had not progressed into the underlying cartilages. No deep ulceration, abscessation or necrotic cartilage (Score 4–5) was identified in this study. Pathological lesions were recorded in 26 of 31 (83%) Texel larynges. The highest score recorded in the study was 3.5 (*n* = 2). Six larynges scored 2.0 and 3.0 respectively. Two scored 1.5. Ten had scores ranging from 0.5–1.0.

#### Relationship between laryngeal anatomy and pathology

A more acute angle between the left cranial point of the cricoid cartilage and the vocal process of the arytenoid cartilage was associated with an increased severity of pathology (*p* = 0.002) (Table 5).. An acute cricoid angle on the right side was non-significant in this model, however when analysed in a separate model with body weight again forced into the model, a similar association was identified, the more acute the angle the greater the pathology score (Table 6).

**Table 5:** Significant associations between dependent and independent variables

Dependent Variable Independent Variable	Coefficient	Confidence Interval (95%)	***P*** Value
**Graded larynx lesion**			
Carcass Weight	−0.12	0.91, −2.18	0.999
Acute angle left	−1.12	−1.82, − 0.45	0.002

**Table 6:** Right angle becomes significant when analysed in a separate model

Dependent Variable Independent Variable	Coefficient	Confidence Interval (95%)	***P*** Value
**Graded larynx lesion**			
Carcass Weight	− 0.12	− 2.38, 2.15	0.917
Acute angle right	−0.78	− 1.48, − 0.069	0.033

## Discussion

No prevalence estimates of laryngeal chondritis (LC) exist nationally or internationally [11]. Surveillance data, however, suggest a likely low prevalence. Murray et al., [15] notes anecdotal suggestions that a small number of flocks in Ireland are experiencing significant within-flock prevalence of laryngeal chondritis. Surprisingly, a high percentage (81.5%) of those surveyed in the current study indicated they previously have had sheep in their flock with ‘throat problems’. It should be noted however, that these are owner-reported data without a definitive diagnosis, as no post mortem examinations were conducted in conjunction with survey respondents reporting ‘throat problems’ to definitively diagnose laryngeal chondritis. While a number of the farmer-diagnosed ‘throat problems’ may be due to pneumonia, dosing-gun injuries, or other respiratory issues, it indicates that further research into laryngeal chondritis in Ireland is warranted.

Laryngeal pathology reported in the current study was predominantly mild oedema, and pathological findings in the current study are much less severe than those reported in other studies [9, 11]. Larynges in the current study were collected at slaughter from animals showing no clinical signs of respiratory disease or LC, as opposed to fallen stock examined in the other studies. Chronic LC is generally regarded as incurable. Early intervention however, has been reported to be successful [6, 14]. The mild lesions reported in the current study may not necessarily have progressed, or indeed resolution may have been possible with early treatment. If cost effective non- invasive ante mortem laryngeal evaluation becomes available in the future, it would be interesting to follow sheep with similarly mild lesions to those reported in this study and assess their progression and the success or failure of therapeutic intervention. While a high proportion of survey respondents in the current study reported successful outcomes following treatment it should again be reiterated that ‘throat problems’ were not definitively diagnosed as laryngeal chondritis in the survey respondents flocks, and the reported cure rates may be due to successful treatment of other respiratory ailments e.g. pneumonia.

A more frequent occurrence of laryngeal chondritis in ewe lambs at the time coinciding with puberty, and shortly after weaning in adult ewes, is reported by Edmunds et al., [16], who also reported that males were generally affected throughout the year. No obvious seasonal pattern was observed by the majority of current study participants. This finding is in contrast with Sigurðardóttir et al., [8], who reported the disease to occur in housed animals predominantly during late winter months. It should be noted however, that the study from Sigurðardóttir et al. [8], was conducted in Iceland where sheep are housed for two thirds of the year due to climate extremes. Interestingly 37.7% of respondents noted ‘throat problems’ more frequently in offspring of a particular sire. While Waine et al., (2019), suggest the anatomy of the Texel sheep may predispose the breed to laryngeal chondritis, this finding warrants further examination as it may suggest the involvement of genetic / hereditary factors in the development of laryngeal chondritis, possibly through influencing laryngeal anatomy, carcass weight or other unknown factors.

Measurements for the current study were performed prior to publication of Waine et al, 2019; therefore there is variation in measurement points. Waine et al, 2019, noted variability in the size and shape of the cricoid ring and first tracheal ring- similar was noted in this study; however as the Waine et al., 2019, study progressed they redid tracheal measurements at the 2-4th tracheal ring and found statistically significant variation in tracheal area. Due to the method of laryngeal collection re-measurement using the methodology of Waine et al was not possible in the current study. Future international studies should aim for a standardised approach for all laryngeal assessment to allow comparison between studies.

In the larynx and trachea, airflow is usually turbulent as the air velocity is high and there is much deposition of inhaled antigens [17]. Turbulent flow driving pressure is proportional to the square of the flow rate, and inversely related to the fifth power of the radius. Therefore, decreases in the radius of the upper airway results in an increase of the driving pressure required to achieve the same airflow [18]. In the current study a more acute angle of the cricoid was found to be significantly associated with increased pathology. Potentially the changes noted in this study and Waine et al., 2019, who noted funneling of the Texel trachea, could contribute to a change in the airway radius, resulting in increased turbulent airflow at the level of the larynx. This turbulent airflow may potentially contribute to trauma of the laryngeal mucosa, allowing deposition of high antigen load onto damaged mucosal epithelium facilitating initiation of chondritis, but this is somewhat speculative and requires additional research.

This study was small in scale and should be repeated on multiple breeds from farms with multiple sires or genetic lines to identify if similar anatomical findings are noted. Additionally both male and female sheep across various age groups and with varying stages of clinical presentations should be examined. As the larynx is a dynamic structure, a weakness of the current study is that it was performed on fixed specimens and future research should examine whether statistically significant measurements reported in the current study can be evaluated ante mortem and identify the most appropriate and cost effective method of assessing larynx structure ante mortem. Additionally as a genetic component has been suggested for the development of laryngeal chondritis, identifying regions of the genome associated with the condition and incorporating this information into a breeding programme would be a further valuable method of producing sheep with less susceptible larynges.

## Conclusion

Respiratory disease was the most frequent problem identified by Texel breeders surveyed in this study. Farmer declared ‘throat problems’ were reported by the majority of those surveyed. Further work involving larynx examination noted laryngeal pathology in Texels showing no clinical signs of respiratory disease. Associations between laryngeal measurements and laryngeal pathology were also identified. Future research should examine whether significant measurements identified in the current study can be measured ante mortem, and whether such ante mortem measurements will allow early identification of sheep at risk of developing laryngeal chondritis.

## Data Availability

The datasets used and/or analysed during the current study are available from the corresponding author on reasonable request.

## Abbreviations

DAFM: Department Agriculture, Food and the Marine
RVL: Regional Veterinary Laboratory
FLE: Fixed Laryngeal Examination
LC: Laryngeal Chondritis

## References

[1] Davenport-Goodall CLM, Parente EJ (2003). Disorders of the larynx. Vet Clin.

[2] Haynes PF, Snider TG, McClure JR, McClure JJ (1980). Chronic chondritis of the equine arytenoid cartilage. J Am Vet Med Assoc.

[3] Milne MH, Barrett DC, Sullivan M, Fitzpatrick JL (2000). Successful medical treatment of laryngeal chondritis in cattle. Vet Rec.

[4] Jeffery ND, Talbot CE, Smith PM, Bacon NJ (2006). Acquired idiopathic laryngeal paralysis as a prominent feature of generalised neuromuscular disease in 39 dogs. Vet Rec.

[5] Caswell JL, Williams KJ, Maxie MG (2016). Respiratory system. Jubb, Kennedy and Palmer's Pathology of Domestic Animals.

[6] Cameron H, Britton JW (1943). Chronic ovine laryngitis. Cornell Veterinarian.

[7] Lane JG, Brown PJ, Lancaster ML, Todd JN (1987). Laryngeal chondritis in Texel sheep. Vet Rec.

[8] Sigurðardóttir ÓG, Jörundsson E, Friðriksdóttir V (2016). Laryngeal Chondritis in sheep in Iceland. J Comp Pathol.

[9] Garrett KS, Embertson RM, Woodie JB, Cheetham J (2013). Ultrasound features of arytenoid chondritis in thoroughbred horses. Equine Vet J.

[10] BJ GP, Lekeux P, Lomba F, editors. Relationship between ventilation mechanics of the larynx and laryngeal disorders in double-muscled Belgian Blue calves. Proceedings of the 14th World Congress on Diseases of Cattle. Dublin: Irish Cattle Veterinary Association; 1986.

[11] Waine K, Strugnell B, Remnant J, Lovatt F, Green M, Rideout H (2019). Anatomy and pathology of the Texel sheep larynx. Vet Sci.

[12] Faull W, Scholes SF. Laryngeal chondritis in texel sheep. Vet Rec. 1987;121(155). 10.1136/vr.121.7.155-c. 3660551

[13] Britton JW (1945). Further observation on chronic ovine laryngitis. Cornell Vet.

[14] Salisbury RM (1956). Chronic ovine laryngitis. N Z Vet J.

[15] Murray GM, Fagan S, Murphy D, Fagan J, Muireagáin CÓ, Froehlich-Kelly R (2019). Descriptive analysis of ovine mortality in sentinel sheep flocks in Ireland. Vet Rec.

[16] Edmunds JL, Roden JA, Finch JM, McEwan NR (2017). Factors affecting the development of laryngeal chondritis in sheep. Large Animal Rev.

[17] Thibeault SL, Rees L, Pazmany L, Birchall MA (2009). At the crossroads: mucosal immunology of the larynx. Mucosal Immunol.

[18] Al-Qadi MO, Artenstein AW, Braman SS (2013). The “forgotten zone”: acquired disorders of the trachea in adults. Respir Med.

